# A new instrument for estimating the survival of patients with metastatic epidural spinal cord compression from esophageal cancer

**DOI:** 10.2478/raon-2014-0043

**Published:** 2015-03-03

**Authors:** Dirk Rades, Stefan Huttenlocher, Amira Bajrovic, Johann H. Karstens, Tobias Bartscht

**Affiliations:** 1 Department of Radiation Oncology, University of Lubeck, Lubeck, Germany; 2 Department of Radiation Oncology, University Medical Center Eppendorf, Hamburg, Germany; 3 Department of Radiation Oncology, Hannover Medical School, Hannover, Germany; 4 Department of Hematology and Medical Oncology, University of Lubeck, Lubeck, Germany

**Keywords:** esophageal cancer, spinal cord compression, metastatic, epidural, irradiation, survival, predictive instrument

## Abstract

**Background:**

This study was initiated to create a predictive instrument for estimating the survival of patients with metastatic epidural spinal cord compression (MESCC) from esophageal cancer.

**Methods:**

In 27 patients irradiated for MESCC from esophageal cancer, the following nine characteristics were evaluated for potential impact on survival: age, gender, Eastern Cooperative Oncology Group (ECOG) performance score, histology, number of involved vertebrae, ambulatory status before irradiation, further bone metastases, visceral metastases, and dynamic of developing motor deficits before irradiation. In addition, the impact of the radiation regimen was investigated. According to Bonferroni correction, p-values of < 0.006 were significant representing an alpha level of < 0.05.

**Results:**

ECOG performance score (p < 0.001), number of involved vertebrae (p = 0.005), and visceral metastases (p = 0.004) had a significant impact on survival and were included in the predictive instrument. Scoring points for each characteristic were calculated by dividing the 6-months survival rates (in %) by 10. The prognostic score for each patient was obtained by adding the scoring points of the three characteristics. The prognostic scores were 4, 9, 10, 14 or 20 points. Three prognostic groups were formed, 4 points (n = 11), 9–14 points (n = 12) and 20 points (n = 4). The corresponding 6-months survival rates were 0%, 33% and 100%, respectively (p < 0.001). Median survival times were 1 month, 5 months and 16.5 months, respectively.

**Conclusions:**

This new instrument allows the physician estimate the 6-months survival probability of an individual patient presenting with MESCC from esophageal cancer. This is important to know for optimally personalizing the treatment of these patients.

## Introduction

In cancer patients, the treatment of loco-regional disease is constantly improving. Therefore, one can expect an increasing number of patients presenting with distant metastases in the future. Bone metastases are quite common in cancer patients and may be associated with complications such as pathological fractures and metastatic epidural spinal cord compression (MESCC).^1^,^2^ Different options are available for treating spinal metastases causing MESCC. These options include decompressive surgery, stereotactic body radiotherapy (SBRT), and different regimens of conventional radiotherapy.^2^ During recent years, personalization of treatment has gained importance, particular for palliative settings and metastatic disease. An optimal personalized treatment approach likely cannot be realized without being able to estimate the patient’s remaining lifespan. Therefore, oncologists have focused more strongly on the development of predictive instruments. Also for MESCC, survival scores have already been developed.^3^ Since each tumor entity leading to spinal metastasis and consequent MESCC has its own biological behavior and meta-static patterns, optimal treatment personalization can only be realized if specific scores are available for each of these entities.^1^,^2^

In the current study, we have created a predictive instrument that allows estimating a patient’s probability to survive at least 6 months following irradiation for MESCC from esophageal cancer.

## Patients and methods

The data of 27 patients irradiated for MESCC were retrospectively analyzed. All patients presented with motor deficits of the legs caused by MESCC. They did not have surgery or irradiation to the involved spinal region before. The diagnosis of MESCC was based on computed tomography or magnetic resonance imaging. Patients were presented to a surgeon prior to irradiation. Dexamethasone was started when MESCC was diagnosed, given during the period of radiation treatment, and tapered down afterwards. Radiotherapy was delivered using a linear accelerator and 6–10 MV photon beams. The treatment volumes encompassed one normal vertebra above and below those vertebrae involved by metastatic disease.

The following nine characteristics were investigated for a potential impact on survival: Age (< 60 years *vs*. ≥ 60 years, median age: 59 years), gender, Eastern Cooperative Oncology Group (ECOG) performance score (1–2 *vs*. 3–4), histology (squamous cell carcinoma *vs*. adenocarcinoma), number of involved vertebrae (1 vertebra *vs*. ≥ 2 vertebrae), ambulatory status before irradiation (ambulatory *vs*. not ambulatory), further bone metastases at the time of irradiation (no *vs*. yes), visceral metastases at the time of irradiation (no *vs*. yes), and the dynamic of the development of motor deficits before irradiation (fast: ≤ 7 days *vs*. slower: > 7 days) (Table 1). Separately, the potential impact of the radiation regimen (short-course: 1 × 8 Gy / 5 × 4 Gy *vs*. longer-course: 10 × 3 Gy / 15 × 2.5 Gy / 20 × 2 Gy) on survival was looked at. For the survival analysis, the Kaplan-Meier method and the log-rank test were used. According to Bonferroni correction for multiple tests, results were considered significant for p < 0.006 representing an overall alpha level of < 0.05. Characteristics achieving a p-value of < 0.006 were included in the instrument developed for estimation of survival. The study was carried out according to the Helsinki Declaration.

## Results

Of the investigated nine characteristic, the following three had a significant impact on survival: ECOG performance score (p < 0.001), number of involved vertebrae (p = 0.005), and visceral metastases (p = 0.004). The results of the survival analysis are presented in Table 2. The additional analysis of the radiation regimen did not reveal a significant association with survival (p = 0.72). Six-months survival rates were 25% after short-course irradiation (3 of 12 patients) and 33% (5 of 15 patients) after longer-course irradiation, respectively. The 12-months survival rates were 8% and 13%, respectively.

**FIGURE 1.**
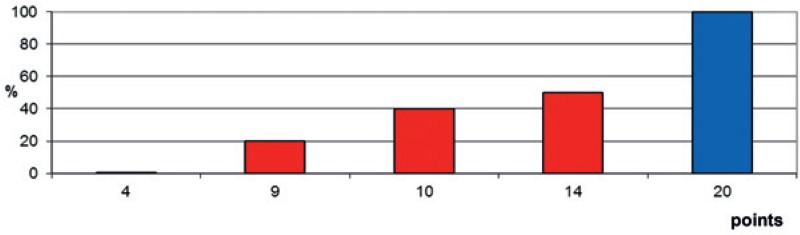
The 6-months survival rates of the prognostic scores (4, 9, 10, 14 or 20 points).

The three significant characteristics were included in the predictive instrument as follows. Scoring points for each characteristic were calculated by dividing the 6-months survival rate (in %) by 10 (Table 3). The prognostic score for each patient was calculated by adding the scoring points of the three significant characteristics. The addition resulted in prognostic scores of 4, 9, 10, 14 or 20 points. The 6-months survival rates of the prognostic scores are shown in Figure 1. Taking into account the 6-months survival rates of the prognostic scores, the following three survival groups were formed: 4 points (n = 11), 9–14 points (n = 12), and 20 points (n = 4). The corresponding survival rates at 6 months were 0%, 33% and 100%, respectively (p < 0.001). Median survival times following irradiation were 1 month (range: 0–3 months), 5 months (range: 2–11 months) and 16.5 months (range: 8–19 months), respectively.

## Discussion

In order to achieve the best results of anticancer therapies, personalized treatment approaches are increasingly used. Individual strategies are particularly important for patients with metastatic disease, since each metastatic situation is quite unique. MESCC is not uncommon in oncology and may occur in up to 10% of adult cancer patients^1^,^2^ “Real” MESCC is associated with neurologic deficits, mostly with motor deficits of the legs. These deficits may range from very mild symptoms to complete paraplegia. Many patients with MESCC are heavily debilitated, whereas others can manage their daily life quite well. In order to optimally tailor the treatment regimen to a patient’s needs, one has to take into account both his current impairment and his remaining lifetime. To choose the most appropriate treatment strategy, it is very important to be able to rate the patient’s survival prognosis as precise as possible. This can be achieved with the application of predictive tools based on prognostic factors. Primary tumors can vary considerably with respect to biological behavior, metastatic spread, response to anticancer treatment, and prognosis. Taking into account these aspects, it becomes obvious that separate predictive tools are important to optimally personalize the treatment. In the present study, we created a survival score specifically for patients with MESCC from esophageal cancer. When using this instrument, the retrospective study design and the relatively small number of patients must be considered. However, since patients with MESCC from esophageal cancer are rare, a larger prospective series will not be available soon.^1^,^2^,^4^

In the current study, three characteristics, ECOG performance score, number of affected vertebrae and visceral metastases, were identified that had a significant impact on survival in such patients. Based on these three characteristics, a predictive instrument including three prognostic groups was developed. The 6-months survival rates of these groups varied considerably. Of the group of patients achieving only 4 points, no patient survived longer than three months. Therefore, these patients are no candidates for decompressive surgery prior to irradiation. They should receive a short course of irradiation, preferably a single fraction of 8 Gy. Several studies have shown that 1 × 8 Gy is not inferior to multi-fraction programs with respect to pain relief and improvement of motor deficits. In a meta-analysis including 5,617 patients from randomized trials, overall pain relief was observed in 60% of patients after single-fraction treatment and 61% after multi-fraction treatment, respectively (p = 0.36).^5^ Complete pain relief was achieved in 23% of patients and 24% of patients, respectively (p = 0.57). in a randomized trial of 276 patients from Italy, 1 × 8 Gy resulted in similar functional outcomes when compared to a longer (two-and-a-half weeks) split-course regimen (3 × 5 Gy followed by one week rest and 5 × 3 Gy).^6^ Sixty-eight per cent and 71% of patients, respectively, were able to walk after irradiation. In a large retrospective study of 1,304 patients from several European countries, 1 × 8 Gy was similarly effective as 5 × 4 Gy in one week, 10 × 3 Gy in two weeks, 15 × 2.5 Gy in three weeks and 20 × 2 Gy in four weeks with respect to improvement of motor function.^7^ Improvement rates at one month after radiotherapy were 26% (1 × 8 Gy), 28% (5 × 4 Gy), 27% (10 × 3 Gy), 31% (15 × 2.5 Gy), and 28% (20 × 2 Gy), respectively (p = 0.90). The post-treatment ambulatory rates were 69%, 68%, 63%, 66% and 74%, respectively (p = 0.58).

Patients achieving 9–14 points in the predictive instrument presented here had a 6-months survival probability of 33% and a median survival time of five months. To be suitable for decompressive surgery, a survival prognosis of at least three months was required in a randomized study from the United States.^8^ Therefore, in selected patients (spinal instability, vertebral fracture, sphincter dysfunction) of this prognostic group the option of surgery followed by longer-course irradiation should be discussed. If surgery is not indicated, these patients should receive fractionated irradiation, for example 5 × 4 Gy or 10 × 3 Gy. One has to be aware that in-field recurrences of MESCC occur more frequently after 5 × 4 Gy than after 10 × 3 Gy.^9^,^10^

Those patients who achieved 20 points in the current score had a favorable survival prognosis of median 16.5 months. All patients survived longer than 6 months. Unfortunately, this prognostic group represented only 15% of the patients in the present study. However, it is important to identify these patients, since they are at a considerably higher risk of developing an in-field recurrence of MESCC than patients achieving ≤ 14 points. MESCC patients with such a favorable survival prognosis were shown in a retrospective study of 382 patients to benefit from 15 × 2.5 Gy or 20 × 2 Gy when compared to 10 × 3 Gy in terms of better local control (risk ratio: 2.42; p = 0.011) and better survival (risk ratio: 1.64; p = 0.014).^11^ Therefore, these patients should receive longer-course irradiation with doses beyond 30 Gy. In addition, these patients should be presented to a surgeon prior to irradiation to discuss whether upfront decompressive surgery is indicated and possible. For highly selected patients, even radiosurgery and SBRT may be considered. When delivering radiosurgery or SBRT, it is mandatory to regard the tolerance doses of spinal cord and vertebral bone, since rates of treatment-related vertebral fractures up to 39% and neurologic deficits up to 8% were reported.^12^,^13^ In an international practice guideline, SBRT was recommended to be used for MESCC only within clinical trials, which was also supported by a recent review article.^14^,^15^

## Conclusions

New predictive instrument has been designed that allows estimating the survival time of patients with MESCC from esophageal cancer. This instrument can assist the physician in selecting the appropriate radiation regimen and in making a decision for or against upfront decompressive surgery.

## Figures and Tables

**TABLE 1.** Characteristics investigated for survival

	**N patients**	**(%)**
Age		
< 60 years	15	56
≥ 60 years	12	44
Gender		
female	5	19
male	22	81
ECOG performance score		
1–2	11	41
3–4	16	59
Histology		
squamous cell carcinoma	11	41
adenocarcinoma	16	59
Number of involved vertebrae		
1 vertebra	6	22
≥ 2 vertebrae	21	78
Ambulatory status before irradiation		
ambulatory	12	44
not ambulatory	15	56
Further bone metastases		
no	9	33
yes	18	67
Visceral metastases		
no	9	33
yes	18	67
Dynamic of developing motor deficits		
fast (≤ 7 days)	10	37
slower (> 7 days)	17	63

ECOG = Eastern Cooperative Oncology Group

**TABLE 2.** Survival rates at 6 and 12 months

	**At 6 months (%)**	**At 12 months (%)**	**P**
Age			
< 60 years	27	7	
≥ 60 years	33	17	0.34
Gender			
Female	40	20	
Male	27	9	0.61
ECOG performance score			
1–2	55	27	
3–4	13	0	<0.001
Histology			
squamous cell carcinoma	27	9	
adenocarcinoma	31	13	0.65
Number of involved vertebrae			
1 vertebra	67	50	
≥ 2 vertebrae	19	0	0.005
Ambulatory status before irradiation			
ambulatory	40	20	
not ambulatory	17	0	0.006
Further bone metastases			
no	56	33	
yes	17	0	0.007
Visceral metastases			
no	67	33	
yes	11	0	0.004
Dynamic of developing motor deficits			
fast (≤ 7 days)	20	10	
slower (> 7 days)	35	12	0.19

According to Bonferroni correction, p-values < 0.006 were considered significant. ECOG = Eastern Cooperative Oncology Group

**TABLE 3.** Survival rates at 6 and the corresponding scoring points

	**Survival rate at 6 months (%)**	**Scoring points**
ECOG performance score		
1–2	55	6
3–4	13	1
Number of involved vertebrae		
1 vertebra	67	7
≥ 2 vertebrae	19	2
Visceral metastases		
no	67	7
yes	11	1

ECOG = Eastern Cooperative Oncology Group
